# Case Title: 45 year-old male with recurrent angioedema: WAO international case-based discussions

**DOI:** 10.1186/1939-4551-7-2

**Published:** 2014-01-22

**Authors:** Jennifer W Leiding, Douglas Beakes, Stephen C Dreskin, Anete Grumach, Michihiro Hide, Avner Reshef, Massimo Triggiani, Michael A Kaliner

**Affiliations:** 1 National Institute of Allergy and Infectious Diseases, National Institutes of Health, Bethesda, MD, USA; 2 Walter Reed National Military Medical Center, Bethesda, MD, USA; 3 University of Colorado, Denver, Aurora, Colorado, USA; 4 Departments of Pediatrics and Dermatology, University of São Paulo Medical School, São Paulo, Brazil; 5 Department of Dermatology, Hiroshima University, Minami-ku, Hiroshima, Japan; 6 Sheba Medical Center, Shoham 97235302605, Israel; 7 Department of Medicine, University of Salerno, Salerno, Italy; 8 Institute for Asthma & Allergy, Bethesda, MD, USA

## 

This month for listing within the *World Allergy Organization Journal* is a paper that represented an innovative experiment first proposed and carried out by Dr. Michael Kaliner. In this paper an interesting case is discussed by a local training program, or programs, in the Washington DC area. The case centers on a particularly difficult subject in allergy management on the subject of angioedema. The original discussion is included, and then comments from expert individuals from five regions of the world were solicited and correlated so that each of the points of view concerning the different regions were registered. This effort took some time to get off the ground, and I believe the initial offering of WAO International Case-based Discussions will generate enough enthusiasm and lead to routine discussions to be published in the *Journal*. The approach invites consideration and comparison of viewpoints from various areas of the world on a specific difficult clinical case.

Again, it was a challenge to pull together. Michael Kaliner and Sofia Dorsano are to be commended for their efforts. As the series expands, it will be quite interesting to follow the different points of view and the regions of the world concerning the common allergy problems we all face.

Lanny J. Rosenwasser, MD

President, World Allergy Organization

## Case report

A 45 year-old Caucasian male presents with approximately 12 episodes of tongue swelling over the last year. Each episode occurs upon awakening in the morning, is preceded by tongue tingling, and is not associated with swelling in other locations of his body. He has no associated airway obstruction but does have difficulty talking and closing his mouth due to the degree of swelling. He has no history of urticaria and does not develop hives in conjunction with tongue swelling. He denies any other signs or symptoms of anaphylaxis.

The patient has been evaluated in the emergency room for many of these episodes and treated with diphenhydramine, prednisone, and ranitidine with improvement in symptoms within an hour of receiving medication. He has also self-treated many episodes with diphenhydramine at home with quick resolution of symptoms. Other times, he has had symptoms of only tongue tingling and is able to abort an episode of swelling with quick treatment with diphenhydramine. He has no history of allergic rhinitis, allergic skin disorders, or food allergy. He has not noticed any specific seasonality, food ingestion, rhinitis, or ocular symptoms associated with episodes.

On review of systems, patient complained of frequent indigestion and heartburn for the last year but denied halitosis, acidic taste in his mouth, or cough. He has no history of mouth sores or ulcers, dental caries, or gingivitis. Laboratory evaluation included normal complete blood count, chemistries, and liver function tests. Thyrotropin was 2.7 mIU/ml (0.27-4.2), C4 25.3 mg/dL (10-40), C1 esterase inhibitor quantity 12 mg/dL (11-26), and C1 esterase inhibitor function >100% (>68%).

After initial evaluation, daily antihistamine therapy was initiated and patient had complete resolution of episodes of angioedema with the exception of one episode after missing two days of therapy. *Helicobacter pylori* IgG was also evaluated due to the patient’s complaints of heartburn. With a positive history and an elevated *H. pylori* IgG at 1.36 u/mL (1-1.09), patient was treated with triple drug therapy including: amoxicillin, clarithromycin, and omeprazole. After therapy for *H. pylori* eradication, patient discontinued treatment of fexofenadine and within 1 day had an episode of angioedema of the tongue. He restarted daily fexofenadine and has remained free of episodes of angioedema.

## International discussion

### Stephen Dreskin (North America)

Idiopathic angioedema is a significant clinical entity that is poorly studied. When a patient presents with episodic swelling in the absence of urticaria, the Allergist/Immunologist first must rule out the bradykinin-mediated angioedemas such as those caused by ACE inhibitors and those due to deficiency of C1 esterase inhibitor [1-3]. The observation, such as in this patient, that symptoms improve on blockade of H1 receptors, suggests that the process is mediated, at least in part, by histamine, a product of mast cells. To produce angioedema in the absence of urticaria, it is likely that the mast cells being activated are in the sub-dermis. So, it is not unreasonable to think of idiopathic angioedema in the same way we think of idiopathic urticaria. The main difference is that in idiopathic angioedema the activated mast cells reside in sub-dermis whereas in idiopathic urticaria they reside in the dermis.

In addition to highlighting the clinical challenge of idiopathic urticaria this case report also raises the issue as to whether infection with *H. pylori* plays a role in chronic urticaria and/or in idiopathic angioedema. This patient was noted to have an elevated *H. pylori* IgG but did not have resolution of his angioedema following treatment designed to eradicate the infection. This raises two related questions: The first is whether there is any reasonable pathophysiologic mechanism to explain an association between infection with *H. pylori* and idiopathic angioedema, and the second is whether there are any clinical data to support this treatment. In regard to the first question, the literature on idiopathic angioedema and chronic idiopathic urticaria has frequently included a potential etiologic role for any chronic infection, possibly acting via immune complex formation or by innate immune pathways.

Regarding clinical data to support such treatment, there are rare case reports to suggest that eradication of *H. pylori* can help idiopathic angioedema [4]. However, given the similarities between chronic urticaria and idiopathic angioedema, is it reasonable to review the evidence that eradication of *H. pylori* can help patients with chronic urticaria? Federman and colleagues performed a meta-analysis of 10 studies (274 subjects) addressing this question with at least modest rigor including evidence that eradication was successful [5]. Overall, successful treatment of *H. pylori* infection was both quantitatively and statistically associated with remission of urticaria with an odds ratio of (OR) = 2.9 (1.4-6.8; p = 0.005) compared to subjects who were *H. pylori* positive without eradication. The British Association of Dermatologists Therapy Guidelines and Audit Subcommittee felt that these data reached a “strength of recommendation level” of “B”, stating there is “fair evidence to support the use of the procedure” [6]. In spite of these data, the utility of examining patients for evidence of *H. pylori* infection is not generally accepted and has been questioned due to the frequency of this infection in asymptomatic individuals [7].

In conclusion, it is reasonable to undertake eradication of *H. pylori* infection in an attempt to modify the natural history of idiopathic angioedema, although the evidence that this would be effective is scant. That said, this case report is missing an important step which is to demonstrate that the eradication of *H. pylori* was successful. This can be accomplished by assaying persistence of *H. pylori* antigen in stool [8].

### Anete Grumach (Central and South America)

It is presented a male patient whose symptoms, mainly tongue swelling, began late, at 45 years old. Genetic disorders usually manifest early in life, for example, hereditary angioedema (HAE). The experience with Brazilian patients has shown that 82% of the HAE patients presented their first symptoms before 10 years old [9].

The tongue was the only location affected, and this is more common for angioedema caused by angiotensin converting enzyme (ACE) in patients treating hypertension [10]. Brazilian HAE patients had facial angioedema in 108 out of 170 but not restricted to the tongue. Allergic angioedema, usually, affects other parts of the body. The patient did not report urticaria or previous history of allergy. On the other hand, several episodes were treated with anti-histamines and steroids, with improvement.

No common triggering factors could be identified by the patient. In our experience with HAE (Brazil; n = 210), trauma was reported by 41.9% (n = 88), followed by stress reported in 26.7% (n = 56). In addition, menses and food were also mentioned in 9.5% and 6.7% of the patients. Considering that ¼ referred stress and approximately 10% did not identify the cause of the attack, it was expected that the patient could not explain the cause of his pain.

Frequent complaints of gastric symptoms were referred, coincident with the initial clinical manifestations in the reported patient. Abdominal pain is the second most frequent symptom reported by HAE patients and epigastric complaints are also common; 43% of HAE patients in our experience. So, the pain referred by the patient could be misdiagnosed as associated with the genetic defect. In contrast, food allergy could be another hypothesis [11].

The gastric manifestation with heartburn directed the physician to the search of gastritis, and the test resulted positive for *H. pylori.* IgG antibodies confirmed the diagnosis. Specific therapy for *H. pylori* improved the occurrence of angioedema, suggesting that this was the triggering factor. Visy et al. (2007) in a collaborative study of seven centers reported the improvement of the frequency of HAE attacks after identifying and treating *H. pylori*[12]. In Brazil, the screening for *H. pylori* in patients with HAE is not a routine. Moreira et al. (2004) found that the occurrence of *H. pylori* in children represents an odds ratio of 4 for developing urticaria [13]. *H. pylori* had also been reported as a worsening factor for Hereditary Angioedema [4,12].

Laboratorial tests excluded hereditary angioedema. Farkas et al. (1999) reported a patient with *H pylori* infection and acquired C1 esterase inhibitor deficiency due to a possible increased consume of complement [14]. This is not the case, because the patient had normal values of complement proteins except for the fact that C1 inhibitor level was close to the lower limit.

### Michihiro Hide (Asia Pacific)

I made a diagnosis of idiopathic or spontaneous angioedema for this patient.

In spite of detailed classifications by the guidelines for urticaria, prepared by EAACI and approved by WAO and several other academic organizations [15], angioedema has still not been well classified, except for subtypes of hereditary angioedema. Recently the Japanese Association of Dermatologists (JDA) revised the guidelines for urticaria and angioedema and classified angioedema into three types: idiopathic angioedema, angioedema due to exogenous factors, and hereditary angioedema [16]. Idiopathic angioedema is a counterpart in angioedema for idiopathic urticaria which was designated in the JDA guidelines. Therefore, this subtype may well be described as *spontaneous angioedema*.

The only one possibility that the author(s) should clearly rule out is the use of angiotensin-converting enzyme inhibitor by this patient [17,18]. The continuous, rather than occasional, use of a drug in this category may reduce the threshold for the appearance of angioedema, which resembles idiopathic urticaria. Namely, patients may intermittently develop angioedema without wheals (superficial and demarcated skin edema) without apparent direct stimuli. However, as far as I know, this type of angioedema is resistant to medication, including anti-histamines. In case the patient described in this report had been taking angiotensin-converting enzyme, he should have first quit this medication.

The association or non-association of wheals with angioedema should be a matter of discussion. The statistical archive reported by Champion about patients with urticaria and angioedema [19], and literature written by many other European and American authors suggest that about half of patients who suffer from chronic idiopathic urticaria, namely wheals, may also develop angioedema [20]. However, this is not applicable to Japanese and probably other Asian patients. I, and many of my colleagues in Japan, have an impression that angioedema and the other subtypes of urticaria are not complicated so frequently. A retrospective study of 260 patients with urticaria and angioedema who visited the outpatient clinic of Hiroshima University Hospital from 2003 to 2005 revealed the population of patients who solely or dominantly suffered from angioedema was 4.2%. Among patients who suffered from chronic spontaneous urticaria (56.2%), only 29 out of 146 patients were complicated with angioedema [21].

A surprising feature of the patient reported here is a good responsiveness to anti-histamines. Oral anti-histamines are the first line medication for idiopathic angioedema but may not completely prevent and suppress symptoms as observed in this report. In a viewpoint of clinical practice, the way of taking medication is important. Unlike spontaneously occurring acute and chronic urticarias, symptoms of angioedema do not appear on a daily basis. Therefore, it might be practical to take medication only when a symptom begins to appear or be felt by the patient. In the case of this patient, it seems to be better to take daily anti-histamine therapy, since the symptoms were severe, and he developed symptoms as early as two days after the cessation of the treatment. It would be a good practice, however, to reduce the amount of anti-histamine and/or prolong the interval of intake to the level where the patient does not develop symptoms. I expect the disease activity of this patient to gradually decrease over the course of treatment and eventually diminish so that he can be free of daily medication.

### Avner Reshef (Middle East/Africa)

This case presentation involves a 45 year-old male with recurrent episodes of isolated tongue swellings, occurring over a period of one year. Most episodes exacerbate upon awakening in the morning, and the patient cannot point at any noticeable triggers, such as foods, medications, stings or infection. These episodes are not accompanied by pruritic rash or signs of systemic anaphylaxis, and seem to respond well to antihistamines and corticosteroids. Laboratory evaluation is negative for C1 esterase Inhibitor (C1INH) deficiency, as the C4 and both antigenic and functional levels of the enzyme are normal. No family members with hereditary angioedema were mentioned. A trial of *H. Pylori* eradication protocol failed to relieve the recurring tongue swelling episodes.

Experienced allergists and ENT specialists and Emergency Department personnel are familiar with this condition, as they are often called to treat and work-up these patients. Angioedema has a wide variety of differential diagnoses that can be mechanistically divided into two major entities: Histamine-mediated swellings are typically allergic or anaphylactic, very often accompanied by intensely pruritic rash (urticaria), while Kinin-mediated edema occurs *silently* and gradually and involve single or multiple sites. In terms of triggers, the earlier condition is associated with exposure to allergens (i.e. foods, stings) or drugs (Aspirin, NSAIDs, ACE Inhibitors), while the other includes hereditary and acquired forms of C1INH deficiency and is triggered mostly by mechanical trauma, infections, and hormones. Rare conditions of tongue swelling and macroglossia include: granulomatous cheilitis (Melkersson-Rosenthal Syndrome), allergic contact dermatitis, lymphedema, lymphangioma, and capillary-leak syndrome.

Zingale et al. recently reported a series of 929 cases of Angioedema without urticaria observed in a tertiary-care clinic in Italy over 11 years period, of which 776 could be evaluated [22]. The face and oral cavity were the most affected areas. Hereditary or acquired angioedema due to C1INH deficiency was diagnosed in 25%. In patients with identified etiology, medications were a possible cause in 45% (mostly ACE Inhibitors), foods in 36%, and only 7% had a concomitant disease (i.e. infection or autoimmune). *H. pylori* infection was evident in two patients, of which only one responded to eradicative therapy. In 38% of cases a cause could not be elucidated, of which 1/3 could be categorized as idiopathic-histainergic edema. Patients with negative laboratory work-up received a long-term antihistamine treatment, for which 86% responded well. A few unresponsive patients improved on tranexamic acid. The authors propose a useful algorithm for the work-up of angioedema without urticaria patients.

I would urge clinicians to extensively investigate patients with recurrent facial or oral cavity angioedema, which can potentially evolve into a disturbing and sometimes life-threatening condition. A careful medication history will sometimes reveal a *forgotten* over-the-counter NSAID taken before bed for a simple headache, or an ACE Inhibitor prescribed for hypertension and skipped by the patient at presentation. A careful work-up by oral specialist will be helpful if local involvement is suspected (MRS, lymphedema, lymphoma).

We have seen in our angioedema clinic a few cases of base-of-tongue and uvular edema in patients with obstructive sleep apnea (OSA) and relentless snoring. We can only speculate on the role of vibratory forces applied by snoring as a cause of local mediator release and vascular hyper-permeability. Laser uvulectomy was helpful in isolated cases.

Therapeutic options for *idiopathic* angioedema without urticaria are still antihistamines (preferentially 2nd generation) and short-term course of corticosteroids. We prescribe tranexamic acid (Hexacapron) 1-1.5 grams b.i.d as a long term prophylaxis regimen for non-responsive patients, particularly where non-histaminergic edema is suspected. The efficacy of the new kinine-system antagonists (icatibant, Firazyre® or ecallantide- Kalbitor®) is presently unclear.

### Massimo Triggiani (Europe)

The case presented can be considered as an idiopathic histaminergic angioedema. This is a diagnosis made by exclusion of other forms of recurrent angioedema such as those clearly related to an allergic reaction or those due to C1 inhibitor deficiencies (hereditary or acquired). Systemic diseases, such as autoimmune, lymphoproliferative or infectious diseases, that can be eventually associated with recurrent angioedema, should have been ruled out by clinical and laboratory findings. Rare forms of angioedema, such as that associated with eosinophilia (Gleich’s syndrome) are not under consideration because of the normal blood count and response to antihistamines. Although no specific information is given whether the patient was on treatment with drugs that can induce angioedema, including ACE inhibitors and ASA, we should expect that the hypothesis of drug-induced angioedema was excluded.

What is really striking in this case is the fact that angioedema apparently occurred always at the same location (the tongue), with no extension to adjacent areas, and at the same time of the day (in the morning). These features may suggest a local factor triggering angioedema and/or a possible exposure to an eliciting factor during the night or early morning. Oropharyngeal infections and previous dental problems, including dental fillings that could cause hypersensitivity, are apparently excluded by the history. In this regard, it would be useful to get information on whether there is occasional use of mouth washings in the morning or whether there is a candidiasis of the tongue. Another possibility that may be taken into diagnostic consideration is a form of *physical* angioedema. Angioedema can be elicited by physical stimuli such as vibration, pressure, and heat. Although urticaria is most frequently related to physical stimuli, isolated angioedema has also been reported and these forms can even be responsive to antihistamines. I would also do tests for physical urticaria/angioedema, and I would check whether there is any association with drinking hot beverages immediately upon awakening.

## Series editor commentary

### Michael A. Kaliner

This article initiates the series of International Case-based Discussions that should appear periodically in this journal hereafter. The idea is to present cases that are discussed from different perspectives, taking advantage of the global presence of the World Allergy Organization. Today’s case is that of recurrent tongue angioedema that was controlled by regular use of oral antihistamines.

While no one provided specific insight into what might be the root cause of this patient’s disorder, it was fascinating to see how the discussions ranged from idiopathic urticaria and angioedema to *H. pylori* to HAE. Unfortunately, no one had a specific proposal for this patient’s problem but all suggested that it had to be histamine initiated (by nature of the effectiveness of oral antihistamines) and thereby suggesting that an allergic mechanism was at play. There is no evidence of either specific antigen sensitization or allergen exposure, so this recurrent syndrome suggests idiopathic mast cell activation of the tongue, perhaps through a non-immunologic mechanism such as a neurogenic stimulus. Many of our angioedema patients have special sites where their swelling occurs more frequently than other sites, but it is uncommon to find a sole site, such as the tongue. One reviewer suggested a possible link to snoring, which is a fascinating idea in this patient.

The idea that *H. pylori* infection might be involved was discussed by most reviewers and the consensus is that the overall evidence weighs against this causative relationship. A possible additional cause might be reflux of gastric fluid overnight into the mouth with specific tongue swelling. Each episode occurred overnight with swelling upon awakening. Every clinician is aware that many allergic subjects awaken and shortly thereafter get sneezing and other nasal manifestations of allergic rhinitis, suggesting that the process of awakening allows these reactions to manifest. It has always seemed unexpected that some subjects can reflux into their throat and nose while in bed in a recumbent position and cause pharyngeal, nasal, sinus, and possible mouth symptoms without being aware of the reflux. However, it would be difficult to explain how this reflux might cause recurrent tongue swelling but no other mouth symptoms.

As with all good case discussions, there are questions remaining without adequate answers; thus the need for expert commentary. This article starts the process of international discussions and we anticipate that there will be more coming in the future issues of the *World Allergy Organization Journal*. We all hope that the readers enjoy this new feature.

## Consent

Written informed consent was obtained from the patient for the publication of this report.

## Competing interest

Dr Grumach declares she has received consulting and teaching fees from Shire, CSL Behring, and FQM, and financial support for research from Shire, and she is on the advisory board on HAE for Shire and Dyax. Dr Hide declares he has received consulting and speaking funds from GlaxoSmithKline, Sanofi-Aventis, and MSD, and speaking support from Japan Boehringer Ingelheim, Tanabe-Mitsubishi E, Shionogi, Kyouwahakkhou-Kirin, Torii, Maruho, Novartispharma, and Shimense Healthcare, and royalties from Shionogi. Dr Reshef declares he has received reimbursements and research funding from Shire HGT, Pharming BV, CSL-Behring AG, and Cephalon-Teva Co. Dr. Leiding, Dr. Beakes, Dr. Dreskin, Dr. Triggiani and Dr. Kaliner declare that they have no competing interests.

## Authors’ contribution

MAK, as series editor, designed the series concept, invited authors, and contributed concluding commentary. JWL and DB, as authors, developed the case report. SCD, AG, MH, AR, and MT reviewed the case and contributed commentary from regional perspectives. All authors and contributors approved the final version of the manuscript to be published.
